# Dysfunctional mitochondrial Ca^2+^ handling in mutant SOD1 mouse models of fALS: integration of findings from motor neuron somata and motor terminals

**DOI:** 10.3389/fncel.2014.00184

**Published:** 2014-07-08

**Authors:** Ellen F. Barrett, John N. Barrett, Gavriel David

**Affiliations:** Department of Physiology and Biophysics, and Neuroscience Program, University of Miami Miller School of MedicineMiami, FL, USA

**Keywords:** mitochondria, motor neuron, motor nerve terminal, Ca^2+^ regulation, mutant SOD1 models of fALS

## Abstract

Abundant evidence indicates that mitochondrial dysfunction and Ca^2+^ dysregulation contribute to the muscle denervation and motor neuron death that occur in mouse models of familial amyotrophic lateral sclerosis (fALS). This perspective considers measurements of mitochondrial function and Ca^2+^ handling made in both motor neuron somata and motor nerve terminals of SOD1-G93A mice at different disease stages. These complementary studies are integrated into a model of how mitochondrial dysfunction disrupts handling of stimulation-induced Ca^2+^ loads in presymptomatic and end-stages of this disease. Also considered are possible mechanisms underlying the findings that some treatments that preserve motor neuron somata fail to postpone degeneration of motor axons and terminals.

## Mitochondria help buffer large stimulation-induced Ca^2+^ loads in motor neurons

Mitochondria are important for temporary buffering of large Ca^2+^ loads in both the soma and peripheral motor terminals of motor neurons. Whole cell recordings using fluorescent Ca^2+^ indicators demonstrate that mitochondria normally buffer about 50% of large Ca^2+^ loads in hypoglossal motor neurons (Fuchs et al., 2013). Imaging studies in motor axons injected with these indicators demonstrate that during repetitive action potential stimulation (e.g., 50 Hz) mitochondrial uptake is also the dominant mode of Ca^2+^ sequestration in motor nerve terminals (reviewed in Barrett et al., 2011). Consistent with this idea, stimulation increases [Ca^2+^] in the matrix of motor terminal mitochondria (David et al., 2003; Vila et al., 2003). Mitochondrial Ca^2+^ uptake is functionally important, because when this uptake is blocked, phasic evoked transmitter release depresses rapidly during repetitive stimulation, likely due to depletion of the vesicle pool by excessive asynchronous transmitter release caused by the increased cytosolic [Ca^2+^] (David and Barrett, 2003).

Mitochondrial Ca^2+^ uptake is especially important in motor neurons that innervate fast, fatiguable (FF) muscles, because these motor neurons tend to have low levels of cytoplasmic Ca^2+^ buffers (e.g., parvalbumin, calbindin, reviewed by Grosskreutz et al., 2010), and tend to discharge in high frequency bursts (Burke, 2004) that deliver large Ca^2+^ loads to motor terminals. Motor neurons that innervate limb FF muscles are especially vulnerable in transgenic mice expressing fALS-associated mutations of superoxide dismutase 1 (SOD1) (reviewed by Gordon et al., 2004; Murray et al., 2010).

**Figure 1:**
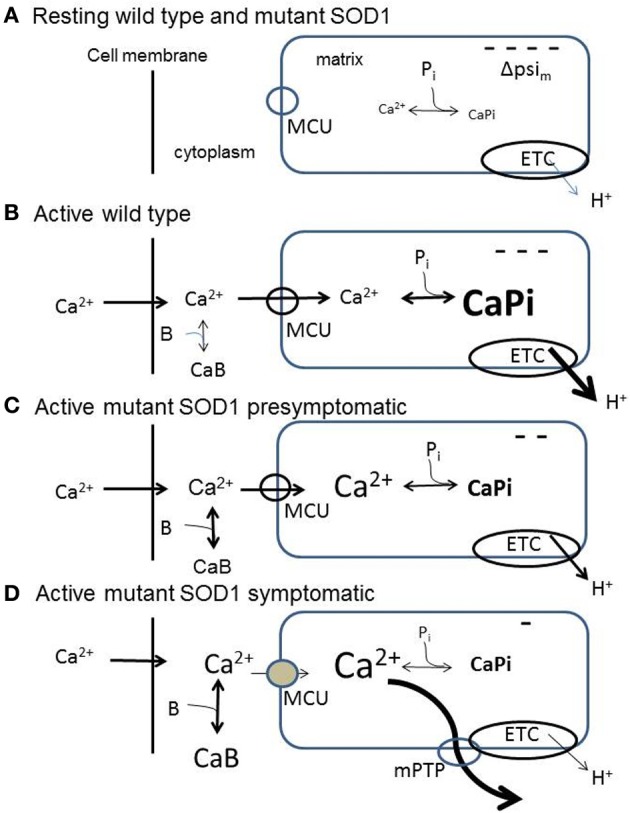
**Diagrams summarizing aspects of mitochondrial handling of stimulation-induced Ca^2+^ loads in wild-type (B) and in presymptomatic (C) and symptomatic (D) SOD1-G93A motor neurons, inferred from measurements in somata and motor terminals. (A)** Resting wild-type and SOD1-G93A motor neurons. ΔΨ_m_ is inside-negative due to H^+^ extrusion by the electron transport chain (ETC). Pi represents dynamic phosphate-dependent matrix Ca^2+^ buffering capacity. **(B)** Wild-type neuron buffering a large stimulation-induced Ca^2+^ influx. At least 50% of this influx enters mitochondria via the mitochondrial Ca^2+^ uniporter (MCU). The mitochondrial Ca^2+^ influx partially depolarizes ΔΨ_m_, thus increasing ETC activity. Matrix Ca^2+^ buffering limits the increase in matrix [Ca^2+^]. **(C)** Presymptomatic SOD1-G93A neuron. Much of the incoming Ca^2+^ load continues to enter mitochondria. Matrix buffering persists, but the ability to accelerate ETC activity is reduced, so ΔΨ_m_ depolarization is greater. **(D)** Symptomatic SOD1-G93A neuron. Total mitochondrial Ca^2+^ uptake is reduced due to (1) greater depolarization of ΔΨ_m_ due to further reduction in the ability to accelerate ETC activity, (2) reduced ability to buffer matrix Ca^2+^, resulting in a greater elevation of matrix [Ca^2+^], even though total mitochondrial Ca^2+^ uptake is reduced. The greater elevation of matrix [Ca^2+^] produces transient openings of the mPTP. In all diagrams, CaB represents non-mitochondrial Ca^2+^ buffering/extrusion (including Ca^2+^ uptake by endoplasmic reticulum), which increases in symptomatic neurons as mitochondrial uptake diminishes. These simplified diagrams omit the outer mitochondrial membrane and the pH-dependence of the matrix Ca^2+^ buffer, and assume that the stimulation-induced Ca^2+^ influx across the cell membrane is not altered by the SOD1-G93A mutation.

Effective sustained buffering of Ca^2+^ by mitochondria requires multiple steps:

1. An elevation of cytosolic [Ca^2+^] sufficient to activate the mitochondrial Ca^2+^ uniporter (MCU, Mallilankaraman et al., 2012). Consistent with the above scenario, mice lacking the MCU have a severely reduced ability to perform strenuous work (Pan et al., 2013).
2. An electrochemical gradient favoring Ca^2+^ entry. The major component of this gradient is the inside-negative membrane potential across the inner mitochondrial membrane (ΔΨ_m_), which favors Ca^2+^ entry (see diagram in Figure 1A). When Ca^2+^ entry depolarizes ΔΨ_m_ (Figure 1B), H^+^ extrusion via the electron transport chain (ETC) accelerates, restoring most of ΔΨ_m_ (Talbot et al., 2007; reviewed in Cozzolino and Carri, 2012). Excessive alkalinization due to increased H^+^ extrusion is prevented in part by electroneutral import of H^+^ by a phosphate carrier, mentioned below. The other contributor to the transmembrane electrochemical gradient for Ca^2+^ is the Ca^2+^ concentration gradient. A powerful dynamic buffering mechanism normally limits increases in free [Ca^2+^] within the mitochondrial matrix to 1–2 μM, even as Ca^2+^ continues to enter mitochondria during repetitive stimulation (David et al., 2003). This buffering occurs because the alkaline matrix pH (created by ETC activity) and phosphate imported into the matrix drive the formation of osmotically-inactive Ca-phosphate complexes (Nicholls and Chalmers, 2004).
3. Mechanisms to extrude matrix Ca^2+^ into the cytosol. In motor nerve terminals a major extrusion mechanism is the mitochondrial Na^+^/Ca^2+^ exchanger (distinct from the plasma membrane Na^+^/Ca^2+^ exchanger) (García-Chacón et al., 2006). More rapid discharge of matrix Ca^2+^ loads can be achieved by opening the mitochondrial permeability transition pore (mPTP), a high-conductance channel in the inner mitochondrial membrane, likely formed by dimers of ATP synthase (see below, Bernardi and Petronilli, 1996; Bernardi and von Stockum, 2012; Giorgio et al., 2013).

## Symptomatic SOD1-G93A motor neuron somata and terminals show reduced mitochondrial Ca^2+^ uptake and transient mPTP opening during stimulation

For technical reasons, to date most studies of mitochondrial function in fALS mice have used mitochondria isolated from (ventral) spinal cord or brainstem, only a fraction of which originate from motor neurons. However, Fuchs et al. (2013) measured mitochondrial contributions to regulation of stimulation-induced elevations of cytosolic [Ca^2+^] in vulnerable hypoglossal motor neurons in SOD1-G93A mice at presymptomatic and end-stage disease. Merging their findings with measurements of stimulation-induced changes in ΔΨ_m_ and matrix [Ca^2+^] from vulnerable motor nerve terminals in mutant SOD1 mice is beginning to suggest mechanisms by which disturbances in mitochondrial function and Ca^2+^ handling develop.

Abnormal mitochondrial morphology is an early event in fALS mice, and some of the earliest detected changes occur in motor terminals (reviewed in Barrett et al., 2011). At presymptomatic ages Nguyen et al. (2009) found that mitochondria in motor nerve terminals innervating a fast muscle depolarize more in response to repetitive stimulation than those in wild-type terminals. These depolarizations were not altered by inhibiting mPTP opening with cyclosporin A (CsA). These findings suggest an early limitation of the ability to accelerate electron transport in response to ΔΨ_m_ depolarization. Damiano et al. (2006) reported reduced ability of ventral horn mitochondria to handle Ca^2+^ loads beginning as early as 65 days in SOD1-G93A mice. A Ca^2+^ imaging study comparing vulnerable presymptomatic motor neurons (hypoglossal, 70 days) to wild-type motor neurons found similar elevations of cytosolic [Ca^2+^] at moderate stimulation frequencies (40 Hz), but greater elevations at maximal stimulation frequencies (Fuchs et al., 2013). In this study a mitochondrial uncoupler significantly impaired Ca^2+^ clearance. Combining information from motor terminals and cell bodies, it appears that at presymptomatic ages vulnerable motor neurons exhibit limitations in mitochondrial responses and Ca^2+^ handling at high frequencies of stimulation, but continue to rely heavily on mitochondria for clearance of large Ca^2+^ loads (Figure 1C).

Disturbances of mitochondrial function and Ca^2+^ handling become much more pronounced as mice become symptomatic and approach end-stage disease (Figure 1D). Nguyen et al. (2011) found that the transient ΔΨ_m_ depolarizations produced by repetitive stimulation were much larger and longer-lasting in end-stage than in presymptomatic terminals. These ΔΨ_m_ depolarizations were reduced by CsA. Vila et al. (2003) reported that in end-stage mice the stimulation-induced elevations of matrix [Ca^2+^] showed upward ramping behavior in contrast to the plateau recorded in wild-type mice. These findings suggest reduced ability to accelerate ETC activity and/or reduced ability to buffer matrix [Ca^2+^]. In addition, the CsA sensitivity and transient nature of the end-stage ΔΨ_m_ depolarizations suggest reversible openings of the mPTP (reviewed in Nicholls, 2009). Temporary reversible openings of the mPTP might provide an alternative, rapid method for removing large Ca^2+^ loads from the matrix (Bernardi and Petronilli, 1996; Bernardi and von Stockum, 2012), acting perhaps as a safety valve. In motor neuron somata of end-stage mice stimulation-induced elevations in cytosolic [Ca^2+^] were no longer affected by mitochondrial uncoupling or blocking the MCU, indicating severely reduced mitochondrial Ca^2+^ uptake, even though there was no change in *resting* Ψ_m_. Expression of cDNAs for MCU-associated proteins was not decreased, but rather increased (Fuchs et al., 2013). These findings are consistent with the idea that mitochondrial defects become apparent mainly when mitochondria are called upon to accelerate ETC activity and/or matrix Ca^2+^ buffering in response to stimulation.

**Figure 2:**
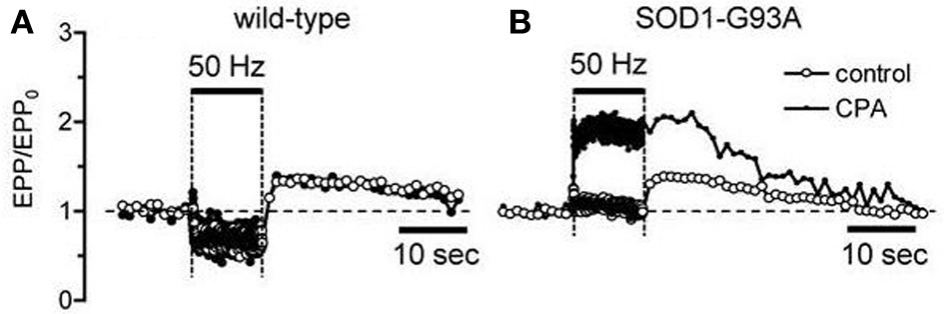
**Inhibiting SERCA pumps increases stimulation-induced changes in end-plate potential (EPP) amplitude in symptomatic SOD1-G93A mice (B) but not in wild-type mice (A)**. Horizontal bars indicate duration of action potential stimulation at 50 Hz. EPP amplitudes, recorded as in David and Barrett (2003), were normalized to their pre-tetanus value (EPP_0_). 50 μM CPA.

When mitochondrial Ca^2+^ handling becomes dysfunctional in end-stage disease, motor neurons must rely more heavily on alternative mechanisms for handling stimulation-induced Ca^2+^ loads. Fuchs et al. (2013) report increased reliance on plasma membrane Ca^2+^ extrusion in end-stage motor neuron somata. Another alternative mechanism is Ca^2+^ uptake into endoplasmic reticulum via SERCA pumps. In wild-type terminals inhibition of SERCA pumps with cyclopiazonic acid (CPA) has no significant effect on stimulation-induced elevations of cytosolic [Ca^2+^] (David and Barrett, 2003) or on stimulation-induced changes in end-plate potential (EPP) amplitude (Figure 2A). However, in symptomatic mutant SOD1 mice CPA greatly increases EPP amplitudes during tetanic stimulation (Figure 2B), suggesting greater elevations of cytosolic [Ca^2+^] when SERCA pumps are inhibited. This increased reliance on non-mitochondrial modes of Ca^2+^ clearance likely subjects cells and terminals to higher peak cytosolic [Ca^2+^] during stimulation, because these non-mitochondrial clearance mechanisms rely on active transport (primary via ATPases or secondary via plasma membrane Na^+^/Ca^2+^ exchanger) and thus may be slower than the rapid passive uptake of Ca^2+^ into mitochondria that becomes possible following opening of the MCU.

## Why does knockout of cyclophilin D (CyPD) preserve motor neuron somata but not motor axons or terminals in mutant SOD1 mice?

In an effort to understand how mitochondrial dysfunction contributes to pathology in mutant SOD1 mice Parone et al. (2013) completed a comprehensive study analyzing the effects of knocking out CyPD, a matrix protein, in mice expressing one of three different fALS-producing SOD1 mutations. CyPD is not required for mPTP formation, but does sensitize the mPTP to Ca^2+^ (Giorgio et al., 2013), so knockout of CyPD would be expected to inhibit mPTP opening by increasing the matrix [Ca^2+^] required for opening. CyPD knockout had many beneficial effects within the spinal cord, including retention of normal mitochondrial morphology, improved mitochondrial ATP synthesis, increased mitochondrial Ca^2+^ buffering capacity, reduced glial activation, reduced aggregation of misfolded SOD1, and improved motor neuron survival. These beneficial effects suggest an important role for mPTP opening in multiple aspects of motor neuron pathology. Nonetheless, the CyPD knockout had no beneficial effect on muscle denervation, motor axon degeneration or disease progression. This result reinforces evidence from multiple studies indicating that this disease includes a dying-back pathology (Fischer et al., 2004; Fischer and Glass, 2007), such that maintenance of the central soma, though necessary, is not sufficient to sustain the motor axon and motor terminal connections with muscle (reviewed by Murray et al., 2010; Dadon-Nachum et al., 2011).

Multiple possible mechanisms might explain why degeneration of motor axons/motor terminals was not postponed by CyPD knockout. Some examples:

1. CyPD knockout may not be sufficient to prevent mPTP opening in motor nerve terminals. Although CyPD sensitizes the mPTP to Ca^2+^, large elevations of matrix [Ca^2+^] can still open the mPTP in the absence of CyPD, and stimulation evokes larger-than-normal elevations of matrix [Ca^2+^] in motor nerve terminals of symptomatic SOD1-G93A mice (Vila et al., 2003). mPTP opening can also be induced by other stimuli whose effects are not influenced by CyPD, including ΔΨ_m_ depolarization and oxidative stress (Bernardi and Petronilli, 1996). These mPTP-inducing stimuli might be especially important in SOD1-G93A motor terminals subjected to the combined stresses of mitochondrial dysfunction and stimulation-induced Ca^2+^ influx, since motor terminals have a much higher surface-to-volume ratio and a higher density of depolarization-activated Ca^2+^ channels than the soma.
2. Inhibition of mPTP opening may not protect motor terminals from degeneration caused by chronic Ca^2+^ overload. CyPD knockout increased mitochondrial Ca^2+^ buffering capacity, but this single aspect of mitochondrial Ca^2+^ handling gives no information concerning the *speed* of Ca^2+^ uptake, a critical determinant of mitochondrial efficiency as buffers for stimulation-induced elevations of cytosolic [Ca^2+^]. Mutant SOD1-induced reductions in the activity of respiratory chain complexes (reviewed in Cozzolino and Carri, 2012) will reduce the rate of mitochondrial Ca^2+^ uptake due to reduced ability to maintain ΔΨ_m_ and reduced dynamic buffering of matrix Ca^2+^, and will also reduce the supply of ATP needed for Ca^2+^ extrusion across the plasma membrane. Thus, mutant SOD1-induced mitochondrial dysfunction might be expected to result in greater and longer-lasting stimulation-induced elevations of cytosolic [Ca^2+^] in motor nerve terminals, with or without mPTP opening.
3. Defective fusion and axonal transport of mitochondria in mutant SOD1 mice (Magrané et al., 2012) might make functional mitochondria scarcer in motor axons and terminals. With fewer functional mitochondria, the metabolic and Ca^2+^-handling loads imposed on each remaining mitochondrion would be greater, increasing the likelihood of chronic Ca^2+^ overload and mPTP opening, as mentioned above. Consistent with this idea, although CyPD knockout preserved mitochondria in motor neuron somata, CyPD knockout did *not* prevent mitochondrial swelling in motor axons (motor terminal mitochondria were not examined, Parone et al., 2013).
4. Some of the mechanisms involved in death may be different for motor axons/ nerve terminals compared to motor neuron soma.
  - While *irreversible* mPTP opening is a trigger for cell death, the *reversible* opening of the mPTP that appears to occur in repetitively stimulated motor terminals of symptomatic SOD1-G93A mice (Nguyen et al., 2011) may serve a beneficial role (e.g., as in heart cells, e.g., Saotome et al., 2009). Temporary mPTP opening might allow the mitochondrion to discharge a dangerously high level of Ca^2+^, then close the mPTP and re-establish Ψ_m_ (reviewed by Bernardi and von Stockum, 2012). Such a mechanism might be especially helpful in motor terminals that experience large Ca^2+^ loads only episodically, as might be expected from the discharge pattern of motor neurons that innervate FF muscle fibers.
  - CyPD knockout may have preserved motor neuron somata by inhibiting a death mechanism specific to the central nervous system. For example, Re et al. (2014) found that astrocytes cultured from ALS patients killed stem-cell-derived motor neurons via necroptosis, a form of programmed necrosis that is Bax-dependent, suggesting mitochondrial involvement.
  - CyPD knockout may fail to prevent disease-related changes in skeletal muscles, which dominate the environment experienced by motor nerve terminals. Skeletal muscles in mutant SOD1 mice evidence signs of hypermetabolism and oxidative stress even at asymptomatic stages of disease (reviewed in Cozzolino and Carri, 2012), and increase expression of Nogo-A, which inhibits formation of neuromuscular junctions (Bros-Facer et al., 2014).
  - Maintenance of motor nerve terminals may require some activity of SOD1 in addition to its dismutase role. Degeneration of motor nerve terminals is one of the earliest events in all fALS-causing SOD1 mutations studied to date (Parone et al., 2013), but the ability of SOD1 mutations to produce motor neuron disease seems unrelated to their dismutase activity (reviewed by Valentine et al., 2005). SOD1 is necessary to maintain neuromuscular junctions, because SOD1 knockout mice gradually lose skeletal muscle innervation, even though motor neuron somata remain intact (Fischer et al., 2012). Perhaps fALS-inducing mutations of SOD1 disrupt this neuromuscular junction-maintaining function via a mechanism independent of dismutase activity.

These considerations suggest that the search for mechanisms underlying ALS pathologies and effective ALS therapeutics needs to include a focus on motor axons, motor nerve terminals and/or skeletal muscle.

### Conflict of interest statement

The authors declare that the research was conducted in the absence of any commercial or financial relationships that could be construed as a potential conflict of interest.
